# Taking Leave During Breast Surgical Oncology Fellowship: A National Evaluation of Policy and Practice

**DOI:** 10.1245/s10434-026-20159-5

**Published:** 2026-07-08

**Authors:** Melissa M. Rangel, Puneet Singh, Andrea Madrigrano, Heather A. Lillemoe, Helen M. Johnson

**Affiliations:** 1 Department of Breast Surgical Oncology, The University of Texas MD Anderson, Houston, TX USA; 2 Department of Surgery, Rush University Medical Center, Chicago, IL USA

**Keywords:** Fellowship, Parental leave, Bereavement, Education, Sick leave, Surveys and questionnaires, Graduate medical education, Clinical competence

## Abstract

**Introduction:**

The American Board of Medical Specialties and the Fellowship Council provide general leave recommendations for many surgical fellowships, yet no standardized policy exists for Breast Surgical Oncology (BSO) programs. Leave is granted on a case-by-case basis by institutions and governing bodies including the Society of Surgical Oncology (SSO) and the American Society of Breast Surgeons (ASBrS). This study characterized the frequency, duration, and types of leave taken by BSO fellows, evaluated institutional leave policies, and barriers to leave implementation.

**Methods:**

An anonymous electronic survey approved by the SSO Research Committee was distributed to program directors and associate PDs of all accredited BSO fellowships in October 2025. Redacted SSO/ASBrS Fellowship Training Committee meeting minutes (2015–2025) were reviewed. Qualitative data on barriers to leave were analyzed using thematic analysis.

**Results:**

The survey achieved a 73% response rate (48/66 fellowships). Two-thirds of responding programs reported a leave policy, and 75% had at least one fellow take leave during training. Review of Training Committee minutes identified 13 fellows who extended training following leave. Maximum leave duration without training extension varied across programs (median 4 weeks, range 0–12) and by type of leave. Thematic analysis identified four recurring barrier themes; the most commonly cited concern surrounded completing fellowship requirements without extending training.

**Conclusion:**

Although leave during BSO fellowship is common, one-third of programs lack a formal policy and durations vary considerably, underscoring the need for a standardized leave framework to ensure equitable trainee experiences.

**Supplementary Information:**

The online version contains supplementary material available at https://doi.org/10.1245/s10434-026-20159-5.

Surgical training is demanding by nature, requiring trainees to balance rigorous clinical and educational obligations with the realities of life outside the hospital. Although trainees are immersed and fully focused on learning to become surgeons, they are not exempt from major life events, such as childbirth, illness, bereavement, and caregiving. Despite this, formal leave policies across surgical training programs are often lacking or are limited in scope and implementation. Among ACGME-accredited primary board specialties, policies are inconsistent and many lack language addressing specific leave types.^1–3^ Furthermore, trainees find existing policies to be inaccessible and inadequate.^2^ Suboptimal leave policies and practices compromise trainee wellbeing and the long-term sustainability of the surgical workforce.^4,5^

In recent years, the adoption of family leave policies among surgical training programs has gained significant momentum and attracted increasing attention. This is likely due, in part, to the evolving surgical workforce. Matriculation of females to medical school has risen from 6.9% to more than 50% in the past several decades, and the proportion of women entering surgical subspecialties continues to grow.^6,7^ As more trainees pursue childbearing and family planning during training, parental leave has emerged as a critical and often unmet need for both birthing and nonbirthing parents alike.^8,9^ With respect to maternity leave specifically, BSO fellowship occupies a notable position among surgical subspecialties, given that women constitute a majority of its trainees, a stark contrast to fields in which female representation remains limited.^10–12^ However, the need for equitable parental leave extends to all fellows regardless of gender or parental role.^10–12^ Beyond parental leave, all trainees share fundamental needs irrespective of sex or family status; bereavement and medical leave are among those most frequently overlooked.^13–16^

The increased attention on leave has resulted in numerous surgical training governing bodies creating formal policies for parental as well as other leave types. In 2022, the American College of Graduate Medical Education (ACGME) mandated a minimum of 6 weeks of paid parental, medical, or caregiver leave.^17^ The American Board of Medical Specialties (ABMS) espouses a similar metric, emphasizing that time away should not disadvantage trainees in board certification.^18^ Following suit, several specialty-specific policies have emerged from governing bodies, such as the American Board of Thoracic Surgery, the Fellowship Council, and the American Board of Surgery (ABS).^19–22^ General leave policy guidance for BSO fellowship training programs exists, but lacks the specificity seen across these other bodies, ultimately leaving discretion up to fellowship program directors and the Society of Surgical Oncology (SSO) and American Society of Breast Surgeons (ASBrS) BSO Fellowship Training Committee (henceforth, Training Committee).^23^ To date, no study has comprehensively evaluated leave policies across BSO fellowship programs. This study primarily aims to characterize the current landscape of BSO leave policies, including parental, medical, and bereavement leave among accredited BSO fellowship programs, with a secondary aim of identifying barriers to implementation.

## Methods

### Survey Development

To collect data on current BSO fellowship leave policies and practices, we developed a survey for distribution to program leaders and requested data from meeting minutes of the Training Committee. Prior to study initiation, a formal written request was submitted to and approved by the SSO Research Committee, the designated body for research oversight within the SSO. Approval encompassed both distribution of the survey instrument and access to redacted SSO fellowship committee meeting minutes.

An internal institutional review board (IRB) determined that this protocol met criteria for IRB exemption.

The survey consisted of three main questions with conditional subquestions based on prior responses, designed to capture both quantitative and qualitative data (Supplementary Fig. 1). Respondents were prompted to report whether their program had a formal leave policy in place and whether a fellow had ever taken leave. Those responding “Yes” to either question were directed to subsequent questions about the number of fellows who had taken leave in the past decade and the maximum number of weeks allotted for leave. Numerical answers were selected using a drop-down menu with integer options ranging from 0–12 as well as a “> 12” option. Responses of “> 12 weeks” were coded as 13 weeks for quantitative analysis. Finally, all respondents were offered a free-text field to describe any obstacles to implementing leave policies. The survey was reviewed by all members of the research group, and multiple rounds of revisions were performed to ensure clarity and content validity. The survey was piloted with a small group of surgeons in education, which led to further refinements. The survey was reviewed and approved by the SSO Research Committee prior to distribution.

### Survey Distribution and Collection

An anonymous electronic survey was distributed by SSO through Qualtrics to all accredited BSO Fellowship Program Directors and Associate Program Directors via email on October 20, 2025, remaining open through November 22, 2025. Two reminder emails were sent at 1 and 2 weeks following initial distribution. The survey was configured to allow only one submission per Internet Protocol (IP) address, and respondents were explicitly instructed to submit a single response per institution.

### SSO Meeting Minute Review and Data Collection

Redacted minutes from the Training Committee meetings were reviewed to capture data on leave requests of any type, including the number of fellows who took leave, leave duration, and any associated training extensions. These meetings served as the forum through which training programs submitted and received approval for leave and training modifications. The review spanned the last 10 years of available minutes (August 2015–August 2025). To protect fellow and program privacy, an SSO staff liaison acted as a neutral third party to redact all identifying information prior to review. Institution- and fellow-specific data were not retained.

### Statistical Analysis

The quantitative survey data were analyzed using descriptive statistical methods. Categorical variables were reported as frequencies and percentages. Continuous variables were reported as measures of central tendency and measures of dispersion. Two authors (MMR and HMJ) independently reviewed the free-text responses describing barriers to leave implementation and performed thematic qualitative analysis. Recurrent themes were identified, grouping responses into categories based on content. Responses of “N/A” were excluded and treated as blank. Themes were subsequently compared across reviewers, and final categories were consolidated through consensus.

## Results

Surveys were sent electronically to the leadership of each of the 66 accredited BSO fellowship programs. There were 48 completed surveys received, representing a response rate of 73%.

### Program Experience with Leave, Formal Leave Policies, and Training Extension

**Fig. 1:**
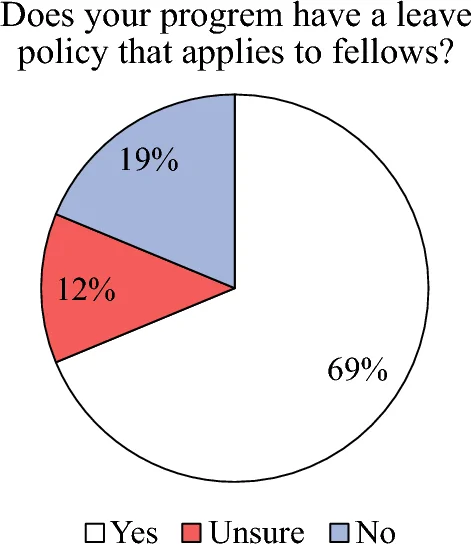
Response to question: Does your institution, department or program have a leave policy that applies to fellows?

**Fig. 2:**
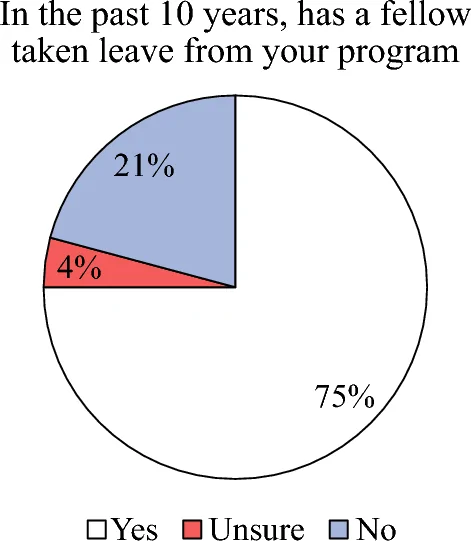
Response to question: In the last 10 years, has your BSO fellowship program had a fellow take leave (medical, personal or parental leave)

When asked whether their institution, department, or program had a formal leave policy applying to fellows, a majority, 33 (69%) reported having a formal policy in place, 19% did not have a policy, and 12% were unsure if a policy existed (Fig. 1). There were 36 programs (75%) that reported having at least one fellow take leave during the past 10 years, 21% did not, and 4% were unsure if this had occurred (Fig. 2).

**Fig. 3:**
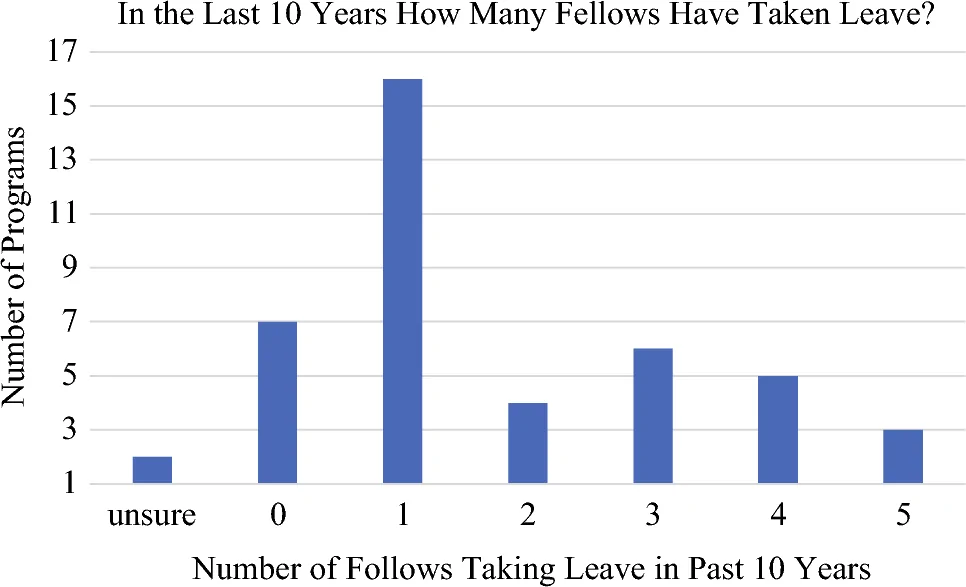
Responses to question: In the last 10 years has your program had a fellow take leave (eg medical, personal or parental leave)?

The distribution of fellows who had taken leave varied across programs. Among the 36 programs that reported leave, 77 total fellows took leave during training. The most common response indicated that 16 programs (44%) had only one fellow take leave, and the greatest number of fellows reported to have taken leave at a single institution was five, reported by three programs (Fig. 3). Seven programs reported never having a fellow take leave, whereas two were unsure if a fellow had ever taken leave. Redacted minutes from the Training Committee meetings were reviewed and showed that 13 fellows extended training after taking leave.

### Program-Reported Leave Types and Duration

**Fig. 4:**
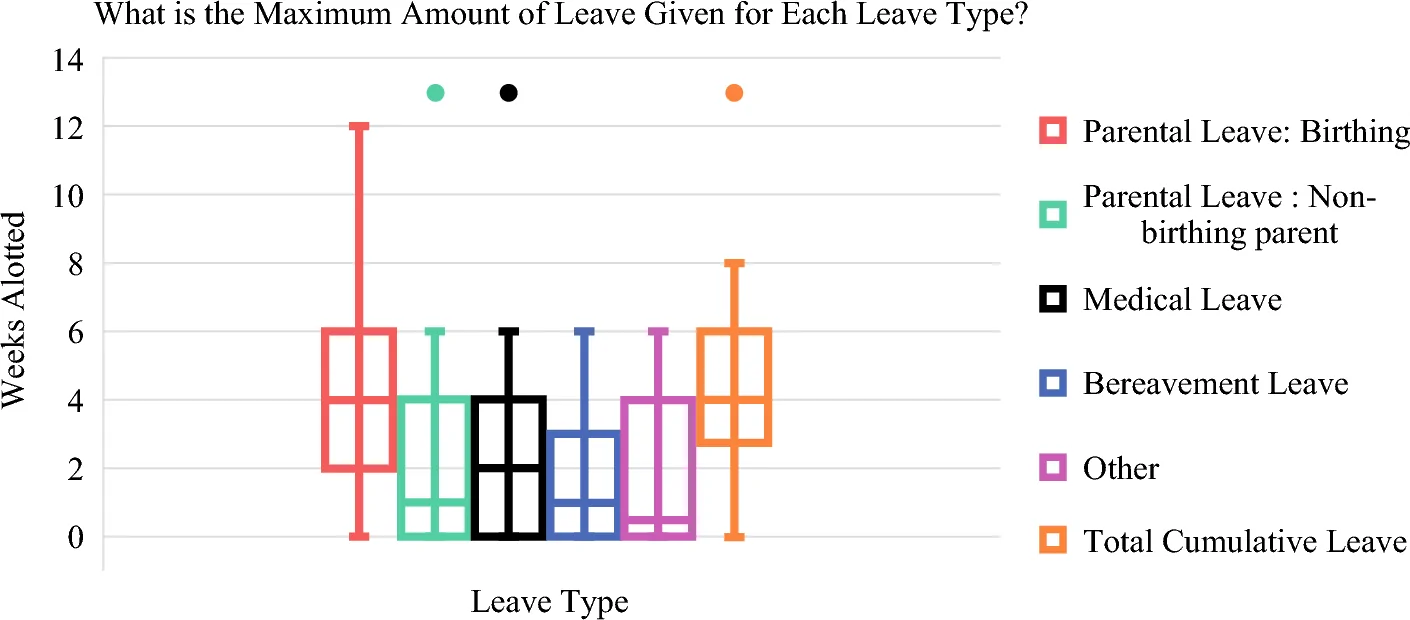
Maximum weeks of leave allowed by leave type without requiring fellowship extension. Box plots display median, interquartile range, and outliers for each leave category

Programs reported leave duration as the maximum number of weeks allowed without requiring a fellowship extension (Fig. 4). The median total leave allotted was 4 weeks (interquartile range [IQR] 3-6). For specific leave types, the greatest duration was parental leave for birthing parents, with a median of 4 weeks (IQR 2–6). Nonbirthing parental leave and bereavement leave were shortest, each with a median of 1 week (IQR 0–4 and 0–3, respectively).

### Barriers to Implementing Leave

A total of 27 free-text responses were collected. Four themes were identified (Table 1). Six responses addressed two themes, yielding 33 total coded answers. The most common theme was “No Obstacles/Issues, Supportive Policies in Place,” reported by 52% (14/27) of respondents, whose comments indicated institutional support for fellows needing leave. The most common barrier-related theme was “Concerns Around Completing Fellowship Requirements, Trainee Competency, and Fellowship Extension,” identified in 41% (11/27) of responses, with comments focusing on difficulties with extending training, financially compensating trainees beyond 12 months, and ensuring operative and clinical competency. “Scheduling/Service Coverage” was reported by 15% (4/27), citing increased burdens on other trainees and advanced care practitioners and gaps in tumor board coverage or other service-related tasks. “Complying with Policy” was also reported by 15% (4/27) of respondents, with descriptions of difficulty navigating competing institutional policies and graduation requirements.

**Table 1:** Program director perspectives on barriers to fellow leave

Theme	Number of responses (*n* = 27*)	Representative examples
No obstacles/issues, supportive policies in place	14 (52)	"None. Our fellowship HR policy has GME oversight, so we followed their protocol and had zero issues with leave"
		"It was not a problem at all. We put her on a research block during her maternity leave"
Concerns around completing fellowship requirements, trainee competency, and fellowship extension	11 (41)	"Can sometimes necessitate extending their fellowship"
		"The biggest challenge is the requirements for the fellowship and guidance from the training committee if time needs to be made up"
Scheduling/coverage challenges	4 (15)	"Required a total reworking of how many of our processes are conducted, including Tumor Board and call"
		"Increases the workload for the residents, APPs, and attendings"
Policy compliance	4 (15)	"Ensuring leave time aligns with SSO fellowship guidelines and GME policies"
		"Payroll and extension of fellowship per SSO/ASBrS guidelines can be tricky given limitations of hospital administrative policies"

Themes identified from survey responses regarding challenges managing parental leave for surgical fellows. *n* = 27*, total coded responses were equal to 33 as some responses addressed multiple themes. Representative quotes are presented verbatim. Numbers presented as frequency (percentage)

## Discussion

This study represents the first comprehensive assessment of leave policies across accredited BSO fellowship programs and demonstrates that while most programs have some formal policy in place, significant variation exists in both the types of leave offered and the duration allowed without extending training. The survey response rate of 73% demonstrates engagement and interest in this topic among program leadership. Qualitative analysis highlighted that while many program directors reported no significant barriers, a substantial proportion expressed concerns regarding fellowship completion requirements, trainee competency, and logistical challenges. Together, these findings suggest an opportunity to develop a common leave structure that supports BSO fellowship programs and their fellows through the many life events that may arise during training.

Our findings reveal significant inequities in leave availability across BSO fellowship programs. Parental leave for birthing parents was reported by 73% of programs (median 4 weeks, IQR 2–6), while nonbirthing parental leave was offered by only 35% (median 1 week, IQR 0–3.5), consistent with patterns observed across surgical specialties where male residents typically take only 1–2 weeks and negative perceptions toward seeking more time persist.^24–27^ This disparity may reinforce gendered assumptions about caregiving and exclude adoptive parents, undermining efforts toward an inclusive training environment. Formal bereavement leave was offered by only 30% of programs, despite evidence that bereavement is among the most traumatic life events, requiring 6–12 months to return to functional baseline, and that trainees are particularly ill-equipped to cope with loss given the demands of fellowship training.^13,14,28^ The current ACGME framework lacks a bereavement standard, leaving programs to inconsistently grant time off and allowing limited time for healthy grief processing.^17^ Medical leave remains similarly nonstandardized. The low utilization of medical leave among BSO fellows as well as the short duration of reported leave is notable given that approximately one-third of surgical trainees have some form of medical disability, yet more than half do not disclose it or seek accommodations.^15,16^ Across all leave types, our results suggest that policy gaps and inconsistent implementation continue to place a disproportionate burden on trainees facing significant life events.

Despite growing recognition of the importance of leave for surgical trainees, common barriers persist among programs. Consistent with published findings from surgical and nonsurgical training programs, program directors in our study most commonly worried about meeting fellowship completion requirements, trainee competency, and training extension.^2,25^ Given that BSO fellowship training spans only twelve months, any period of leave represents a substantial proportion of the total training experience, raising legitimate concerns regarding competency and the ability to meet graduation requirements. Scheduling and coverage challenges have also been widely reported.^2,25,29,30^ Importantly, however, decisions regarding leave duration and structure should be guided by the educational needs of the fellow rather than by service coverage considerations. Proposed solutions such as hiring advanced practice providers or utilizing moonlighting coverage have been suggested, although these require departmental funding and institutional support beyond the program level.^25,30^ Taken together, our findings indicate that barriers to leave reflect both structural and cultural challenges embedded in surgical training more broadly.

Recommendations for leave during fellowship range from specific to quite broad, with room for interpretation. ACGME and ABMS have established policies recommending 6 weeks of leave without impact on vacation time or training duration, although the ABMS policy does not apply to BSO fellowships as it was written for certain 2-year programs.^17,18^ More than a dozen different types of 1-year fellowships exist, operating under a wide array of specific governing bodies, with leave guidelines ranging from no formal policy to a maximum of 8 weeks.^31–33^ The Fellowship Council oversees the largest group of 1-year fellowships, governing 186 surgical programs. The Fellowship Council allows for a maximum of 8 weeks of leave during a 1-year program, with a minimum of 44 weeks of clinical activity required for on-time graduation, while acknowledging that local institutional policies may differ.^20^ BSO fellowships are not governed by any of these bodies, however, and leave is considered an individual programmatic decision without specified time or graduation requirements.^23^ With this amount of variability leave policies, it is unsurprising that a recent needs assessment found only a third of trainees knew how to access parental leave policies, and a survey of complex general surgical oncology and BSO fellows found nearly 90% could not identify any formal governing policy, with graduation requirements identified as the most influential factor in determining leave duration.^24,34^ Our data complement these findings, suggesting existing guidelines have not yet translated into consistent access to leave across fellowship programs. Centralizing leave parameters and graduation requirements, as the Fellowship Council has modeled, may help to address this gap by establishing a single, accessible resource for trainees and a clear framework to guide programs in their planning. Standardization of this kind has the potential to reduce variability and ensure that fellows across all program types are afforded the same protections.

Addressing these gaps will require more than policy mandates. A recent longitudinal review found a steady rise in trainee-reported life events including childbirth, bereavement, and medical emergencies, underscoring the need for institution-specific strategies that support the whole trainee beyond isolated leave events.^35,36^ Our findings, taken together with the broader literature, suggest closer evaluation of programmatic implementation is warranted to ensure equitable access to parental, bereavement, and medical leave without penalizing fellows' training trajectories. Fellowship leave policies and practices must balance two equally legitimate interests: the completeness of surgical training and the holistic wellbeing of the fellow. Indeed, the downstream consequences of inadequate training—for the surgeon and their future patients—are profound and must not be minimized in discussions of leave policy. At the same time, actively supporting and ensuring trainee wellbeing is an ethical imperative. A well-designed leave framework should not force a choice between these priorities, but instead preserve educational integrity while ensuring fellows are not penalized for unavoidable life events.

This study has several limitations. As a survey-based study, responses are subject to recall bias, and program directors may have relied on memory when reporting the frequency and duration of leave taken by fellows over the preceding decade or not have been present at the institution over that entire duration. Several measures were implemented to prevent duplicate responses. We nonetheless acknowledge that existence of duplicate responses cannot be entirely excluded. Furthermore, this study did not capture data regarding the financial implications of fellow leave or the extent to which funding structure influences programmatic decision-making around leave policy. Understanding these dynamics, including the potential budgetary impact of extended training duration, represents an important area for future investigation. It should also be noted that in an attempt to protect anonymity of programs, this study did not collect data regarding program type (e.g., academic, independent) or size, nor did it capture information on the presence of other learners or advanced practice providers. These factors may impact logistical considerations and play a meaningful role in shaping programmatic decision-making around leave policy. Finally, response bias is an inherent limitation of survey-based research; programs with existing formal leave policies or those with leadership already invested in advancing trainee education and wellbeing may have been more likely to participate, potentially overestimating the prevalence of structured leave policies across all BSO fellowship programs.

## Conclusions

Leave remains inconsistently offered across BSO fellowship programs and disproportionately favors parental leave for birthing parents, with program directors citing fellowship completion requirements as a significant obstacle to leave implementation. These findings are consistent with broader patterns documented across surgical training disciplines. Notably, the Training Committee has recently incorporated two flexible weeks into the revised BSO fellowship curriculum, providing programs with additional opportunities to possibly accommodate leave without compromising requirements or extending training.^37,38^ While policy existence alone does not guarantee the ability to implement desired changes, a more standardized leave framework may create an opportunity for BSO fellowships to ensure all fellows, regardless of gender, family structure, or circumstance, have access to the time and support they need during major life events, without jeopardizing their training or career trajectory.

## Supplementary Information

Below is the link to the electronic supplementary material.

Supplementary file1 (PDF 390 KB)
