# Supplementary material for: Taking Leave During Breast Surgical Oncology Fellowship: A National Evaluation of Policy and Practice
Source: Ann Surg Oncol. 2026 Jul 8;33(10):8729–35. doi: 10.1245/s10434-026-20159-5 (PMC13585786; doi:10.1245/s10434-026-20159-5)
Supplement: Supplementary file 1 [file 10434_2026_20159_MOESM1_ESM.pdf]

QUESTION 1
Leave History & Policy

In the last 10 years, has your BSO fellowship program had a fellow take leave (eg medical, personal or parental leave)

- ◯ Yes
- ◯ No
- ◯ Unsure

Does your institution, department or program have a formal leave policy that applies to fellows?

- ◯ Yes
- ◯ No
- ◯ Unsure

QUESTION 2
Number of Fellows on Leave

In the last 10 years how many fellows have taken leave?

Select one

- Select one
- Unsure
- 0
- 1
- 2
- 3
- 4
- 5
- 6
- 7
- 8
- 9
- 10
- 11
- 12
- >12

QUESTION 3
Maximum Leave Duration by Type

For each of the following types of leave, what is the maximum number of weeks fellows in your program are allowed to take without extending their fellowship? This should not include vacation time.

Parental leave: birthing parent

Select one

Parental leave: non-birthing parent

Select one

Medical leave

Select one

Bereavement leave

Select one

Other personal leave

Select one

Total cumulative leave

Select one

QUESTION 4
Obstacles & Challenges

Please describe any obstacles that prevented a fellow from taking leave or challenges the program faced once the fellow was on leave.
